# P376: Dirty bedpans and MDRO: partners in crime?

**DOI:** 10.1186/2047-2994-2-S1-P376

**Published:** 2013-06-20

**Authors:** GG Van Knippenberg-Gordebeke

**Affiliations:** 1 Knip Consultancy Infection Prevention, Venlo-Boekend, The Netherlands

## Introduction

In 2006 the International Organization for Standardization (ISO) published Standard 15883 for Washer-disinfectors (WD), with Part 3 for human waste containers: what states that manual procedures must be avoided as much as possible. I conducted research to get insight in the awareness of ISO15883 and into bedpan management.

## Objectives

Awareness rising for safe bedpan management.

## Methods

Conducting a Global survey (2010) of bedpan management, the awareness of ISO15883 and bedpans causative for HAI. I performed Planned and ‘secret shopper‘ visits in hospital sluice rooms (31) on all continents and had Experience exchange with colleagues (>250).

## Results

Survey

- Response:13% in 54 countries

- HAI (4-21%) reported with: *Clostridium difficile*, *Norovirus*, *MRSA*,

- *Pseudomonas aeruginosa*, Salmonella species and *Acinetobacter baumannii*

- Majority never searched for bedpans as a potential source

- Manual handling is done (65%)

- ISO15883 is known in northwest Europe (76%) in the rest of the world (14-37%)

Visits

- Diapers and urine catheter as replacement for bedpans is regular done

- Nurses, nurse aids, relatives and others do the job without training ‘how to’

- Personal Protective Equipment is seldom used

- Bedpans without covers are transported to a toilet or slobhopper

- Manual empty of the content with (bloody) faeces causing splashes and splatters

- Regular only ‘cleaning’ by rinsing or spraying with cold water

- Disinfection without attention for the right product, duration and prior cleaning

- Not well equipped sluice rooms

- Macerators or WD are not maintained or validated

Experience exchange

Nurses and physicians seldom look after the process and are glad that it is brought to their attention. Most WD or macerators are often malfunctioning or unused. No available guidelines for bedpan management.

## Conclusion

There is a risk for HAI caused by poorly performed bedpan management. The risk of handling bedpans can be easily ignored because the chosen practice depends on habits, rituals and budget. Manual handling contributes to the risk of environmental contamination and transmission. In case of using machines maintenance and validation must be regular checked. Hospitals have to recognize their weakness in bedpan management and must set improvements and investments.

## Disclosure of interest

G. Van Knippenberg-Gordebeke Consultant for For MEIKO Germany
